# Analysis of Clinical Features and Outcomes of Infective Endocarditis with Very Large Vegetations: A Retrospective Observational Study from 2016 to 2022

**DOI:** 10.31083/j.rcm2308264

**Published:** 2022-07-21

**Authors:** Xiaoyun Cheng, Jie Meng, Yanqiu Chen, Fan Zhang

**Affiliations:** ^1^Department of Pulmonary and Critical Care Medicine, Xiangya Hospital of Central South University, 410000 Changsha, Hunan, China; ^2^Hunan Key Laboratory of Organ Fibrosis, 410000 Changsha, Hunan, China; ^3^Department of Pulmonary and Critical Care Medicine, The Third Xiangya Hospital of Central South University, 410000 Changsha, Hunan, China; ^4^Department of Anesthesiology, Xiangya Hospital of Central South University, 410000 Changsha, Hunan, China

**Keywords:** infective endocarditis, vegetation, operative indication, surgical timing

## Abstract

**Background:**

Cases of infective endocarditis (IE) with >30 mm vegetations are rare and are associated with high mortality. Clinical experience, clear therapeutic standards, and outcome evidence about these cases are still lacking.

**Methods:**

Detailed clinical data from patients suffering from IE complicated with >30 mm vegetations were collected from a hospital medical record system. Age- and sex-matched IE cases with 10–20 mm vegetations were used as a control group.

**Results:**

Twenty-two patients with >30 mm IE vegetations confirmed by biopsy and transthoracic echocardiography (TTE) were included. Thirteen (59.0%) patients had basic cardiac diseases, mainly congenital heart disease (CHD), rheumatic heart disease, and device-related issues. Fever (81.8%), heart murmur (86.4%), heart failure (86.4%), and embolism (50.0%) were common clinical manifestations and complications. TTE showed the diameter of vegetations was 34.5 (30.0–39.8) mm. The vegetations were usually accompanied by severe valvular regurgitation and pulmonary hypertension, and were most often located in the mitral valve (38.4%). Laboratory examinations indicated anemia, hypoalbuminemia, heart failure and inflammation. The rate of positive blood culture was 68.2%. Streptococcus viridans was the most frequent pathogen (26.7%). All individuals underwent vegetectomy and valve replacement or repair surgery, within 2 days of diagnosis. Compared with 10–20 mm vegetations group, >30 mm vegetations group had more complicated basic cardiac diseases, more special microbial infection, higher levels of procalcitonin (PCT) and D-dimer, more common heart failure and embolism. They received more biological valve replacements, and had longer intensive care unit length of stay (ICU-LOS). A few patients developed significant postoperative adverse events, including intracerebral hemorrhage (ICH), septic shock, and new symptomatic thrombosis. Re-exploratory thoracotomy was performed in two cases. All patients survived during 6-month follow-up without IE recurrence in >30 mm vegetations group, while there was one death and one recurrence in the 10–20 mm vegetations group.

**Conclusions:**

For IE complicated with >30 mm vegetations, clinical characteristics are diverse and vegetations on TTE are prone to misdiagnosis as thrombus or tumors. This article also emphasizes the use of >30 mm IE vegetations as an independent indication for early surgery to improve prognosis.

## 1. Introduction 

The number of endocarditis episodes diagnosed worldwide in one year could be as high as 250,000 cases [1], and the mortality rate within one year exceeds 30%. Especially when large vegetations form, often with multiple severe complications, the situation is often life-threatening, requiring early diagnosis and timely intervention.

Vegetations of infective endocarditis can form on the native or prosthetic heart valve. Vegetations >10 mm are often treated, making very large vegetations rare in clinical practice and in the literature [2]. The clinical and imaging features of infective endocarditis (IE) with large vegetations are different from those of typical IE [3]. Very large heart vegetations are prone to misdiagnosis as thrombus or tumor, causing clinical difficulties in the diagnosis. It is very important to supplement the clinical characteristics of IE patients with very large vegetations [4]. Alarmingly, the high bacterial density and fragility of massive vegetations usually lead to severe IE complications, valve destruction, heart failure, vessel embolism, sepsis, and immune phenomena [5]. Furthermore, the size of vegetations is associated with in-hospital mortality [6]. However, the guidelines issued by the American College of Cardiology (ACC) and the European Society of Cardiology (ESC) have uncertainty and variation among surgical indications, risks, and timing for >30 mm infective valvular vegetation, and these recommendations are limited by the low level of evidence from primary observational studies [7]. In summary, the experience in diagnosis, treatment and management of >30 mm vegetation has been largely lacking [8]. Therefore, this study retrospectively analyzed the detailed clinical data and prognosis of 22 patients with IE complicated with >30 mm vegetation in our hospital from January 2016 to February 2022. We also retrieved and summarized all IE case reports with >30 mm vegetations published between 2020 and 2022. The aims are to improve clinicians’ understanding of IE with very large vegetation and to provide evidence for clinical practice.

## 2. Materials and Methods

### 2.1 Participant Selection and Clinical Information 

Twenty-two patients with IE complicated with large vegetation were included in the study. They were diagnosed by ultrasound, intraoperative observation, pathogen and postoperative biopsy results according to modified Duke’s criteria [9]. Data include age, gender, risk factor such as basic heart diseases, signs and symptoms (fever, dyspnea, cardiac murmur, other signs of embolism, etc.), laboratory indicators (white blood cells [WBC], hemoglobin, neutrophil percentage [NE%], lymphocyte percentage [LY%], C-reactive protein [CRP], erythrocyte sedimentation rate [ESR], procalcitonin [PCT], N-terminal pro-brain natriuretic peptide [NT-proBNP], D-dimer, blood culture, drug sensitivity test results, etc.), transthoracic echocardiography (TTE), treatment, comorbidity, and outcomes. Twenty-two age- and sex-matched IE cases with 10–20 mm vegetations were used as a control group.

### 2.2 Evaluation Tools and Definitions

The main analysis method was comparative analysis shown in the tables and a tornado diagram. The highest body temperature from the onset of the initial symptom until surgery and blood test results at admission were recorded. Surgical risk was evaluated using the established European System For Cardiac Operative Risk Evaluation II (EuroSCORE II) [10]. The search strategy for all case reports (2020 to 2022) of IE with >30 mm vegetation follows Christopher Radcliffe’s article [2].

### 2.3 Statistical Analysis

Data were processed with SPSS 26 (IBM, Armonk, NY, USA). Enumeration data are shown as n (%), and were compared with the chi-square tests between the two groups. Considering that the sample size is relatively small due to the rarity of cases, all measurement data are expressed in median (25th–75th percentiles [Q1–Q3]) and were analyzed using nonparametric tests. Preoperative WBC count, NE%, CRP, ESR, prothrombin time (PT), fibrinogen degradation product (FDP), activated partial thromboplastin time (APTT), direct bilirubin, NT-proBNP, lactate dehydrogenase (LDH), creatine kinase (CK), PCT, D-dimer were compared with the upper reference limit, while hemoglobin, LY%, total protein, albumin were compared with the lower limit of the reference value. The single-sample Wilcoxon test was conducted to compare patients’ laboratory indicators with reference values. The Wilcoxon paired rank test was used for preoperative and postoperative comparisons. Quantitative data comparing the >30 mm vegetations group and the 10–20 mm vegetations group was assessed with two Independent Samples Nonparametric Test. A *p*-value < 0.05 was considered statistically significant.

## 3. Results

### 3.1 Baseline Characteristics and Risk Factors

Basic information and clinical characteristics of the 22 patients with >30 mm IE vegetations (mean age 45.0 ± 15.6 years; 45.5% males) and the 22 patients with 10–20 mm vegetations are presented in Table 1. The two groups were comparable. In the >30 mm IE vegetations group, the average EuroSCORE II was 4.2 ± 2.8. Most (59.0% (13/22)) patients had at least one basic heart disease, among which congenital heart disease (CHD) was predominant, accounting for 27.3% (6/22). CHD included two cases of mitral valve prolapse, and one case each of ventricular septal defect, bicuspid aortic valve, double aortic arch (DAA), and aortic sinus aneurysm. CHD was followed in frequency by three cases with previous rheumatic heart disease that had undergone prosthetic valve replacement, one case of aortic valve prolapse and two cases of cardiac device-related infective endocarditis (CDRIE), which were related to a Cardiac Resynchronization-Defibrillator Device (CRTD) and a ventricular demand pacemaker (VVI) respectively. Additionally, there was one case each of dilated cardiomyopathy (DCM) and one of hypertrophic cardiomyopathy (HCM). Other risk factors included recent skin infection (two cases), diabetes (two cases), and stage 5 chronic kidney disease (CKD5) (one case). In the 10–20 mm group, the basic condition of the heart in 11 cases (50.0%) was CHD, including mitral valve prolapse (5/22), bicuspid aortic valve (3/22), ventricular septal defect (VSD), and one case each of aortic valve prolapse and patent ductus arteriosus. One patient had a history of tooth extraction, one had a history of drug abuse, and two had tonsil infections.

**Table 1.** **Baseline characteristics of the study patients (n = 22) and age- and sex-matched controls (n = 22)**.

Characteristics		>30 mm group	10–20 mm group	*p* value
Demographics				
Male sex		10 (45.5)	11 (50.0)	0.763
Age (years)		41(33–59)	52 (31–52)	0.324
	≤30	4 (18.1)	6 (27.3)	-
	30–60	12 (54.5)	13 (59.1)	-
	>60	6 (27.3)	3 (13.6)	-
EuroSCORE II		4 (3–5)	3 (2–4)	0.062
Risk factors				
Basic heart disease		13 (59.0)	11 (50.0)	0.545
CHD		6 (27.3)	11 (50.0)	0.122
Previous rheumatic heart disease		3 (13.6)	0	-
CDRIE		2 (9.1)	0	-
HCM		1	0	-
DCM		1	0	-
Recent skin infection		2 (9.1)	0	-
Diabetes		2 (9.1)	1	-
CKD5		1	1	-
Manifestations				
Fever		18 (81.8)	14 (63.6)	0.176
Maximum body temperature (°C)		38.9 (37.8–39.7)	38.1 (37.2–39.1)	0.252
Dyspnea or chest pain		10 (45.5)	6 (27.3)	0.210
Cardiac murmur		19 (86.4)	21 (95.5)	0.294
Arrhythmia		9 (40.1)	4 (18.2)	0.099
Recent Complications				
Heart failure		19 (86.4)	10 (45.5)	0.004*
Embolism		11 (50.0)	6 (27.3)	0.122
	cerebral infarction	6 (27.3)	3 (13.6)	
	Spleen	4 (18.2)	3 (13.6)	
	limb vessel	2 (9.1)	0	-
	Pulmonary embolism	2 (9.1)	0	-
	Others	4 (18.2)	1	

Continuous variables are presented as median (Q1–Q3), counts as n (%). Abbreviations: EuroSCORE II, European System for Cardiac Operative Risk Evaluation II; CHD, congenital heart disease; CDRIE, cardiac device-related infective endocarditis; HCM, hypertrophic cardiomyopathy; DCM, dilated cardiomyopathy; CKD5, chronic kidney disease stage 5. *p < 0.05.

### 3.2 Symptoms and Signs

**Fig. 1.**
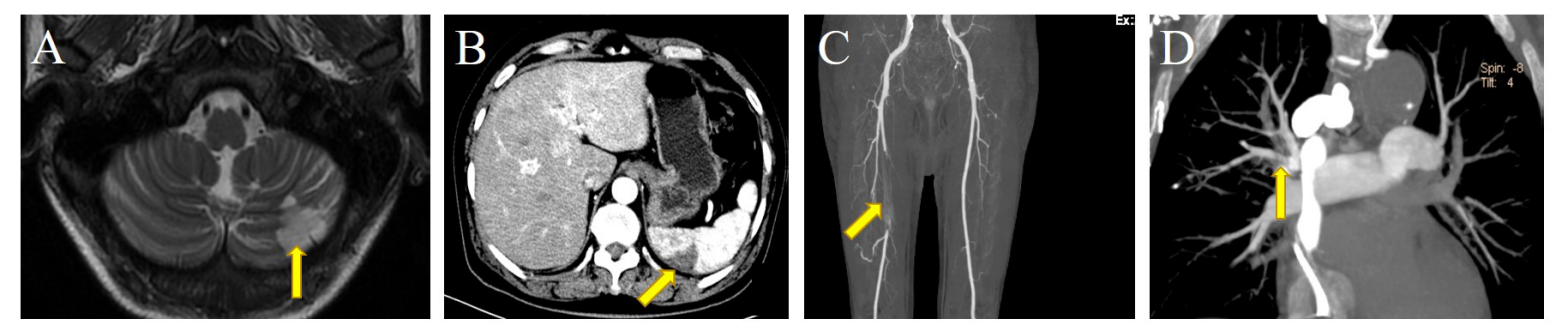
**Embolization of different sites in patients in this study**. (A) Arrow: left cerebellar hemisphere infarction. (B) Arrow: spleen infarction, manifesting as subcapsular wedge-shaped reduced density of the spleen. (C) Arrow: the right femoral artery occlusion, with filling defect. (D) Arrow: pulmonary embolism.

In the >30 mm IE vegetations group, fever (18 cases, 81.8%) was a prominent clinical manifestation with an average maximum value of 38.9 (37.8–39.7) °C. Other symptoms included dyspnea in nine cases, chest pain in one case, and cough in one case. The most common signs were cardiac murmur in 19 cases (86.4%) and arrhythmia in 9 cases (40.1%), followed by splenomegaly in 4 cases (18.2%). Osler nodes and Janeway lesions were uncommon. The complications mainly comprised cardiac insufficiency (19 cases, 86.4%) and embolization (11 cases, 50.0%) (Table 1). Embolization was in the brain (six cases), the spleen (four cases), a limb vessel (two cases), a pulmonary vessel (two cases), a mesenteric vessel (one case), the kidney (one case), the liver (one case), and the superior vena cava (one case) (Fig. 1). Compared with the 10–20 mm vegetations group, heart failure was more common in patients with >30 mm vegetations (86.4% vs. 45.5%, *p* = 0.004), and embolism was also more common, but without a statistically significant difference (50.0% vs. 27.3%, *p* = 0.122).

### 3.3 Laboratory Examinations 

#### 3.3.1 Basic Laboratory Tests 

In the >30 mm IE vegetations group, laboratory analysis (Table 2) revealed anemia, hypoproteinemia, inflammation and heart failure. Laboratory findings exhibited statistically significant decreases in hemoglobin (89.5 [72.0–98.5] g/dL, *p *< 0.001), LY% (15.5 [8.9–18.5], *p *< 0.001), and albumin (31.2 [27.9–35.1] g/L, *p *< 0.001). Indicators above the upper reference limit were CRP (61.9 [32.3–83.4] mg/L, *p *< 0.001), ESR (67.0 [34.8–82.5] mm/h, *p *< 0.001), PCT (1.5 [0.2–2.4] ng/mL, *p *< 0.001), NT-proBNP (1686 [966.1–3535.0] pg/mL, *p *< 0.001), LDH (336.0 [217.7–403.5] U/L, *p* = 0.013). Liver enzymes and serum creatinine were grossly normal. Coagulation function suggested hypercoagulability, with D-dimer (0.7 [0.4–1.4] mg/L, *p* = 0.024) exceeding the upper limit of the reference value. Cardiac troponin I (cTn I) was also statistically elevated compared with its reference value (0.1 [0.0–0.2] ng/mL, *p* = 0.043). PCT and D-dimer in the >30 mm vegetations group were significantly higher than those in the 10–20 mm group. Other laboratory indicators showed no statistical differences (**Supplementary Table 1**). In the >30 mm vegetations group there was transient deterioration of some postoperative indicators, including inflammatory indicators (Preoperative value vs. postoperative value: PCT: 1.5 [0.2–2.4] vs. 2.3 [1.2–5.4], *p* = 0.017), blood coagulation (PT: 13.8 [12.9–14.9] vs. 14.7 [13.8–21.9], *p* = 0.026. D-dimer: 0.7 [0.4–1.4] vs. 1.1 [0.7–3.0], *p* = 0.019) and cTn I: 0.1 [0.0–0.2] vs. 1.5 [0.4–2.2], *p* = 0.005, NT-proBNP: 1686 [966.1–3535.0] vs. 3458 [1168.0–10979.0], *p* = 0.012, CK: 28.9 [26.7–59.0] vs. 66.5 [27.4–269.0],* p* = 0.034) after surgical manipulation, and gradually resolved without specific therapeutic intervention. Kidney and hepatic function showed no remarkable change compared with their preoperative levels.

**Table 2.** **Preoperative and postoperative laboratory variables in blood tests**.

Laboratory variables	Preoperative value	Reference value	*p* value (vs. reference)	3rd post-operative day	*p* value (vs. postoperative value)
WBC count (×${\mathrm{10}}^{9}$/L)	9.4 (7.4–13.4)	3.5–9.5	0.615	13.2 (11.6–15.3)	0.006†
Hemoglobin (g/L)	89.5 (72.0–98.5)	130.0–175.0	<0.001*	88.0 (76.8–102.8)	0.851
Platelets (×${\mathrm{10}}^{9}$/L)	191.0 (97.5–276.3)	125.0–350.0	NA	209.0 (118.0–263.0)	0.963
NE (%)	80.2 (69.6–87.5)	40.0–75.0	<0.001*	85.9 (82.0–89.1)	0.108
LY (%)	15.5 (8.9–18.5)	20.0–50.0	<0.001*	7.0 (5.2–11.6)	0.029†
CRP (mg/L)	61.9 (32.3–83.4)	<8.0	<0.001*	76.7 (41.0–105.0)	0.243
ESR (mm/h)	67.0 (34.8–82.5)	<21.0	<0.001*	72.3 (47.5–86.0)	0.508
APTT (s)	31.8 (22.6–29.5)	25.0–43.0	NA	35.8 (29.5–42.0)	0.265
Albumin (g/L)	31.2 (27.9–35.1)	40.0–55.0	<0.001*	31.2 (26.9–35.1)	0.935
TBIL (μmol/L)	12.8 (9.5–17.9)	1.7–17.1	NA	10.6 (7.6–19.6)	0.639
DBIL (μmol/L)	6.1 (4.2–10.7)	<6.8	0.783	5.8 (3.4–9.6)	0.581
Creatinine (μmol/L)	111.5 (78.5–128.0)	<111.0	NA	68.6 (62.0–99.0)	0.622
LDH (U/L)	336.0 (217.7–403.5)	120.0–250.0	0.013*	391.0 (280.3–461.6)	0.203
Total protein (g/L)	74.7 (62.4–74.7)	65.0–85.0	0.733	65.1 (54.2–70.0)	0.935
PT (s)	13.8 (12.9–14.9)	10.0–16.0	NA	14.7 (13.8–21.9)	0.026†
Urea (mmol/L)	4.4 (2.8–9.1)	2.6–7.5	0.465	5.7 (4.3–9.0)	0.445
NT-proBNP (pg/mL)	1686 (966.1–3535.0)	<125.0	<0.001*	3458 (1168.0–10979.0)	0.012†
ALT (U/L)	23.6 (15.3–36.5)	7.0–40.0	NA	19.7 (13.6–35.2)	0.494
AST (U/L)	24.6 (18.0–39.6)	13.0–35.0	0.277	29.6 (20.7–53.1)	0.117
CK (U/L)	28.9 (26.7–59.0)	40.0–200.0	0.845	66.5 (27.4–269.0)	0.034†
CK-MB (U/L)	11.4 (7.2–16.9)	<24.0	NA	14.9 (10.3–24.8)	0.569
PCT (ng/mL)	1.5 (0.2–2.4)	<0.05	<0.001*	2.3 (1.2–5.4)	0.017†
D-dimer (mg/L)	0.7 (0.4–1.4)	<0.5	0.024*	1.1 (0.7–3.0)	0.019†
cTn I (ng/mL)	0.1 (0.0–0.2)	<0.04	0.043*	1.5 (0.4–2.2)	0.005†

Variables are presented as median (Q1–Q3). NA (not applicable): that the laboratory index was basically normal, which is significantly lower than the high reference value and significantly higher than the low reference value. Abbreviations: WBC, white blood cells; NE%, neutrophils percentage; LY%, lymphocytes percentage; CRP, C-reactive protein; ESR, erythrocyte sedimentation rate; APTT, activated partial thromboplastin time; TBIL, total bilirubin; DBIL, direct bilirubin; LDH, lactate dehydrogenase; PT, prothrombin time; NT-proBNP, N-terminal pro-brain natriuretic peptide; ALT, alanine aminotransferase; AST, aspartate aminotransferase; CK, creatine kinase; CK-MB, creatine kinase MB; PCT, procalcitonin; cTn I, cardial troponin I. *p < 0.05 vs. reference. †*p *< 0.05 vs. the value on 3rd post-operative day.

#### 3.3.2 Blood Culture and Drug Sensitivity 

**Fig. 2.**
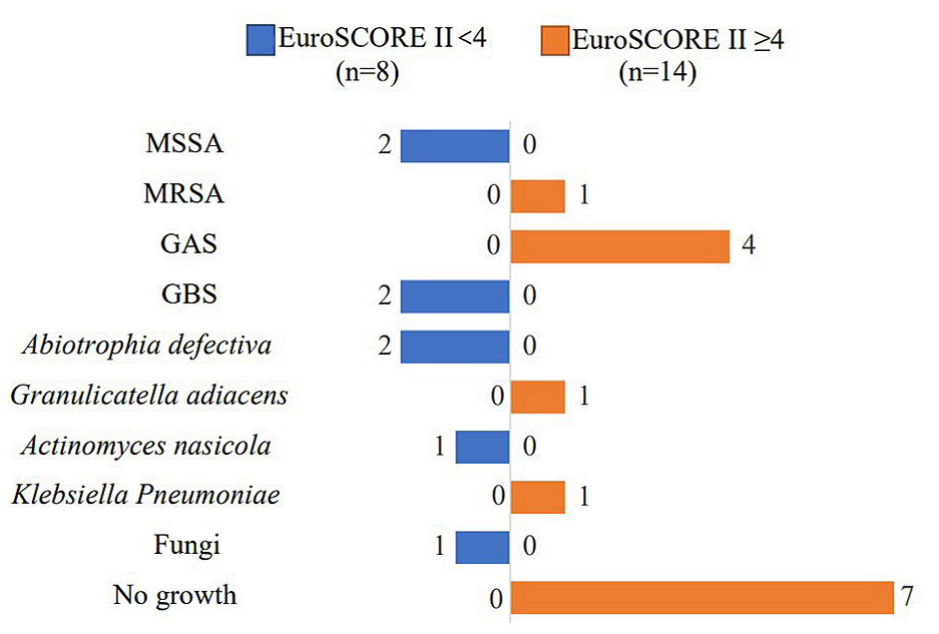
**Acquisition of infective endocarditis and causative organisms (N= 22)**. Blue: EuroSCORE II <4 group. Orange: EuroSCORE II ≥4 group. Abbreviations: MSSA, methicillin-sensitive *staphylococcus aureus*; MRSA, methicillin-resistant *staphylococcus aureus*; GAS, group A streptococci; GBS, group B streptococci.

All 22 patients got blood cultures more than two times. Fifteen patients were positive in blood culture. We found *Streptococcus* in six cases, *Staphylococcus aureus* in three cases, and one case each of *Abiotrophia defectiva*, *Actinomyces nasicola*, *Klebsiella pneumonia*, *Granulicatella adiacens*, and *Aspergillus*. Interestingly, in the EuroSCORE II <4 group (n = 8), there were two cases of methicillin-sensitive *Staphylococcus aureus* (MSSA), and two cases of group B streptococci (GBS), while group A streptococci (GAS), Methicillin-resistant *S. aureus* (MRSA), Carbapenem-resistant *Enterobacter* (CRE) and negative culture conditions were only observed in EuroSCORE II >4 group (n = 12) (Fig. 2). Seventy-five percent (9/12) of gram-positive bacteria were sensitive to ampicillin (AMP), 50% (6/12) were sensitive to Vancomycin (VAN), and 50% (6/12) were sensitive to Gentamicin (Table 3). In the 10–20 mm vegetations group, there were 12 cases of positive blood culture, 11 cases of *Streptococcus* (*S. sanguis*, *S. parahemococcus*, *S. gordon*, *oral S. mutans*, *S. thoraltensis*, *S. pharyngitis*), and one case each of *Faecococcus* and *Enterococcus* lead.

**Table 3.** **Antibiogram from gram positive bacteria in the study patients (N = 12)**.

Antibiotics	Sensitive pathogen				10–20 mm group	
	*Staphylococcus aureus* (n = 3)	*Streptococcus* (n = 6)	*Abiotrophia defectiva* (n = 2) and *Actinomyces nasicola* (n = 1)	Total (N = 12)	*Streptococcus* (n = 10)	Total (N = 12)
Ampicillin	0	6	3	9	10	12
Oxacillin	2	0	0	2	0	0
Vancomycin	3	0	3	6	10	11
Gentamicin	3	0	3	6	0	1
Ceftriaxone	0	3	3	5	10	12
Rifampicin	3	2	0	5	0	0
Meropenem	0	3	1	5	10	12

### 3.4 Echocardiography 

The ultrasonic diagnosis was consistent with the pathological diagnosis in 81.8% (18/22) patients. One case was misdiagnosed as thrombosis and three cases were misdiagnosed as atrial myxomas. The average length of the vegetations was 36.9 ± 7.4 mm (range: 31–59 mm). Vegetations often appeared as moderate to slightly high echo areas, most commonly located in the mitral valve (eight cases, 38.4%), with fewer in the tricuspid valve (five cases, 22.7%) and in the aortic valve (four cases, 18.2%) (Table 4). Most vegetations were mobile (20 cases, 90.9%), and were blocky (16 cases, 72.7%) or had strip structure (six cases, 27.3%). In terms of complications, TTE revealed that there were severe valvular regurgitations in 15 cases (68.2%) and pulmonary hypertension in eleven cases (50.0%). In 10–20 mm vegetations, there were 20 cases of vegetations in the left heart and 2 cases in the right heart, including 1 case in the pulmonary valve and right ventricular outflow tract. Similar to the >30 mm group, multiple, clumpy vegetations and severe valve regurgitation (11 cases) were also common. But patients with pulmonary hypertension were fewer than those with >30 mm vegetations (18.2% vs. 50.0%, *p* = 0.026).

**Table 4.** **TTE manifestation in this study**.

The traits of vegetations	>30 mm group	10–20 mm group	The lesion of vegetations	>30 mm group	10–20 mm group
Maximum diameter (mm)	34.5 (30.0–39.8)	12.5 (11.0–15.0)	Mitral valve	8 (36.4)	10 (45.5)
Multiple vegetations	12 (54.5)	16 (72.7)	Aortic valve	4 (16.7)	8 (36.4)
Crumby structure	16 (72.7)	13 (59.1)	Tricuspid valve	5 (22.7)	1 (4.5)
Strip structure	6 (27.3)	9 (40.9)	Mitral and aortic valve	4 (16.7)	2 (9.1)
Pulmonary hypertension	11 (50.0)	4 (18.2)	Tricuspid and aortic valve	1 (4.5)	0 (0.0)
Severe valvular regurgitation	15 (68.2)	11 (50.0)	EF value (%)	53.0 (49.0–57.0)	59.0 (55.8–64.0)

Variables are presented as count (%) or median (Q1–Q3).

### 3.5 Treatment and Clinical Outcomes

All 22 patients received antimicrobials and surgical treatment. Patient age, sex, intensive care unit length of stay (ICU-LOS), locations of vegetations, antimicrobial therapy, pathogen, EuroScore II, surgical treatments and major complications are summarized in Table 5. All 22 patients received initial empiric antibiotic therapy, and then physicians individually adjusted the antibiotics regimen according to the etiological evidence. Fourteen patients (63.6%) were treated with two or more antibiotics. Ten patients (45.5%) received cephalosporins-based antibiotic-treatment, often combined with Piperacillin-Tazobactam (TZP), VAN, and AMP. The most commonly used antibiotic was cephalosporin (in 14 cases), followed by penicillin (in 5 cases), and VAN (in 3 cases).

**Table 5.** **Treatment regimen and outcomes of 22 IE patients**.

	Age/Sex	ICU-LOS (days)	Major involved area	Antimicrobial Therapy (duration before surgery)	Pathogen	Euro Score II	Surgery	Major complications
**Native valve involvement**								
1	29/M	<1.0	Mitral annulus	OXA (1 d)	MSSA	0	MVP + TVP	Septic shock
2	54/M	<1.0	PML	CRO (5 d)	*S. sanguinis*	5	MVR	-
3	54/F	3.0	AML, PML	CRO/TZP (5 d)	*S. agalactiae*	11	MVR + TVP	-
4	25/F	<1.0	PML	CRO (6 d)	*Abiotrophia defectiva*	0	MVR	-
5	17/F	10.0	PML	CRO/TZP (1 d)	MSSA	3	MVR	ICH
6	62/F	1.9	PML	CRO (2 d)	No growth	11	MVR	Cardiac arrest
7	37/F	<1.0	AV	OXA/CRO/TZP (8 d)	*S. intermedius*	4	DVR	ICH
8	52/M	<1.0	AV	CRO (1 d)	MRSA	3	DVR	SP
9	50/F	3.0	AV	CRO (1 d)	*Actinomyces naeslundii*	2	AVR	-
10	54/M	<1.0	BAV	CXM (3 d)	No growth	4	AVR + DAA operation	-
11	52/M	2.0	MV + AV	AMP/GEN (3 d)	*Abiotrophia defectiva*	4	AVR	Septic shock
12	75/F	1.8	MV + AV	CRO/TZP (8 d)	*Granulicatella adiacens*	8	Bentall + MVR	Hemoperi-cardium, SP
13	45/F	3.5	MV + AV	CRO/VAN (4 d)	No growth	4	AVR + PFO closure	-
14	53/M	<1.0	ATVL	AMP/CZO (3 d)	No growth	4	TVR	-
15	64/F	4.8	ATVL	IMP/TZP (19 d)	*S. sanguinis*	5	CRT-D extraction	PE, DIC
16	34/M	<1.0	TSL	AMP/CRO (8 d)	*S. oralis*	5	TVR + aortic aneurysm repair	PE
17	36/F	1.7	ATVL	VOR/MFX (5 d)	*Aspergillus flavus*	0	TVR	Multiple atrial thrombus
18	66/M	6.8	MV + ATVL	CFP/VAN/IMP (14 d)	*Klebsiella Pneumoniae*	9	TVR + AVR	-
19	37/M	6.9	AML	VAN/CRO (1 d)	No growth	6	MVP + TVP	-
**Prosthetic valve involvement**								
20	62/F	<1.0	AML	CFP (2 d)	No growth	6	MVR	-
21	66/F	5.4	PML	CXM/VAN (14 d)	No growth	5	DVR + TVR	Pericardial thrombus, ICH
22	29/F	<1.0	ATVL	VAN (6 d)	*S. agalactiae*	3	TVR	-

Abbreviations: ICU-LOS, intensive care unit length of stay; AML, anterior mitral leaflet; PML, posterior mitral leaflet; AV, aortic valve; BAV, bicuspid aortic valve; MV, mitral valve; ATVL, anterior tricuspid valve leaflet; OXA, oxacillin; CRO, ceftriaxone; TZP, piperacillin-tazobactam; CXM, cefuroxime; AMP, ampicillin; GEN, gentamicin; VAN, vancomycin; CZO, cefazolin; IMP, imipenem; VOR, voriconazole; MFX, moxifloxacin; CFP, cefoperazone; MRSA, methicillin-resistant *staphylococcus aureus*; MSSA, methicillin-sensitive *staphylococcus aureus*; MVP, mitral valve repair; TVP, tricuspid valve repair; DVR, double valve replacement; AVR, aortic valve replacement; DAA, double aortic arch; PFO, patent foramen ovale; CRT-D, cardiac resynchronization-defibrillator device; TVR, tricuspid valve replacement; ICH, intracerebral hemorrhage; SP, severe pneumonia; PE, pulmonary embolism; DIC, disseminated intravascular coagulation.

**Fig. 3.**
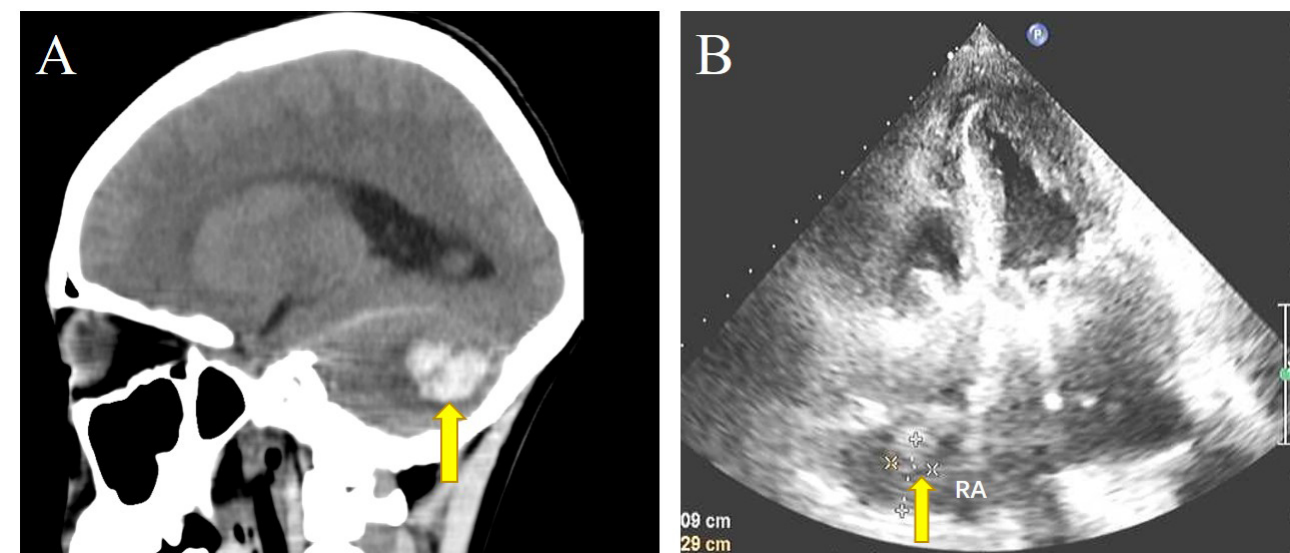
**Postoperative adverse events**. (A) The patchy high-density foci in the left cerebellar hemisphere of Case. 21, and the plain computed tomography (CT) value was about 72 Hounsfield units. Arrow: left cerebellar hemorrhage. (B) Arrow: local thrombus in the pericardium under the roof of the right atrium.

Most (86.3%, 19 cases) patients had at least one valve replaced, and most (72.7%, 16 cases) underwent surgery within one week of admission. Intraoperative observation found leaky or faulty valves in 16 cases, including 5 cases of valve rupture, 1 case of paravalvular abscess, and 1 case of valve perforation. Except for one patient who only underwent cardiac resynchronization-defibrillator device (CRT-D) extraction, all patients received valve replacement or repair and vegetectomy surgery, with vegetations collected for pathological biopsy. Patients were transferred to cardiac surgery intensive care unit (ICU) after surgery, with a median of length of stay (LOS) of 1.75 days (range: 0.42–6.9 days), and a median hospital stay of 16.5 days (range: 8–48 days). Severe postoperative adverse events after surgery were as follows: ICH in three cases (bleeding sites were subarachnoid space, frontal lobe and cerebellum (Fig. 3)), and septic shock in two cases. Other adverse events included severe pneumonia (SP), pulmonary embolism (PE), and disseminated intravascular coagulation (DIC). Case 6 lost consciousness twice, and suffered from cardiac arrest. A temporary pacemaker was installed because of post-sinus arrest. She underwent surgery again on the tenth day after surgery due to sternal dehiscence. Case 12 developed postoperative hypovolemic shock and electromechanical separation. On the 3rd day after operation, thoracotomy was performed again to reveal hemopericardium and to stop the bleeding. Case 21 developed local thrombosis in the right atrium after the operation, leading to restricted right atrial filling (Fig. 3). As his blood indicators improved, he was discharged with oral warfarin 1.25 mg daily. All 22 patients survived without IE recurrence during six-month follow-up. Only one patient developed heart failure requiring hospitalization for his previous dilated cardiomyopathy. In patients with 10–20 mm vegetations, 14 patients (63.6%) received a single antibiotic (6 cases of second-generation cephalosporin, 4 cases of ceftriaxone (CRO), and 4 cases of TZP), and patients with enterococci received penicillin + levox/cef-benzacillin. At least one valve was replaced in 86.3% (19 cases), and 90.1% (20 cases) underwent surgery within one week of admission. The average intensive care unit length of stay (ICU-LOS) was 21.0 (15.5–45.3) hours. One patient developed postoperative hemorrhage in the right temporal lobe with a hematoma volume of 40 mL, and died one week after discharge.

## 4. Discussion

**Fig. 4.**
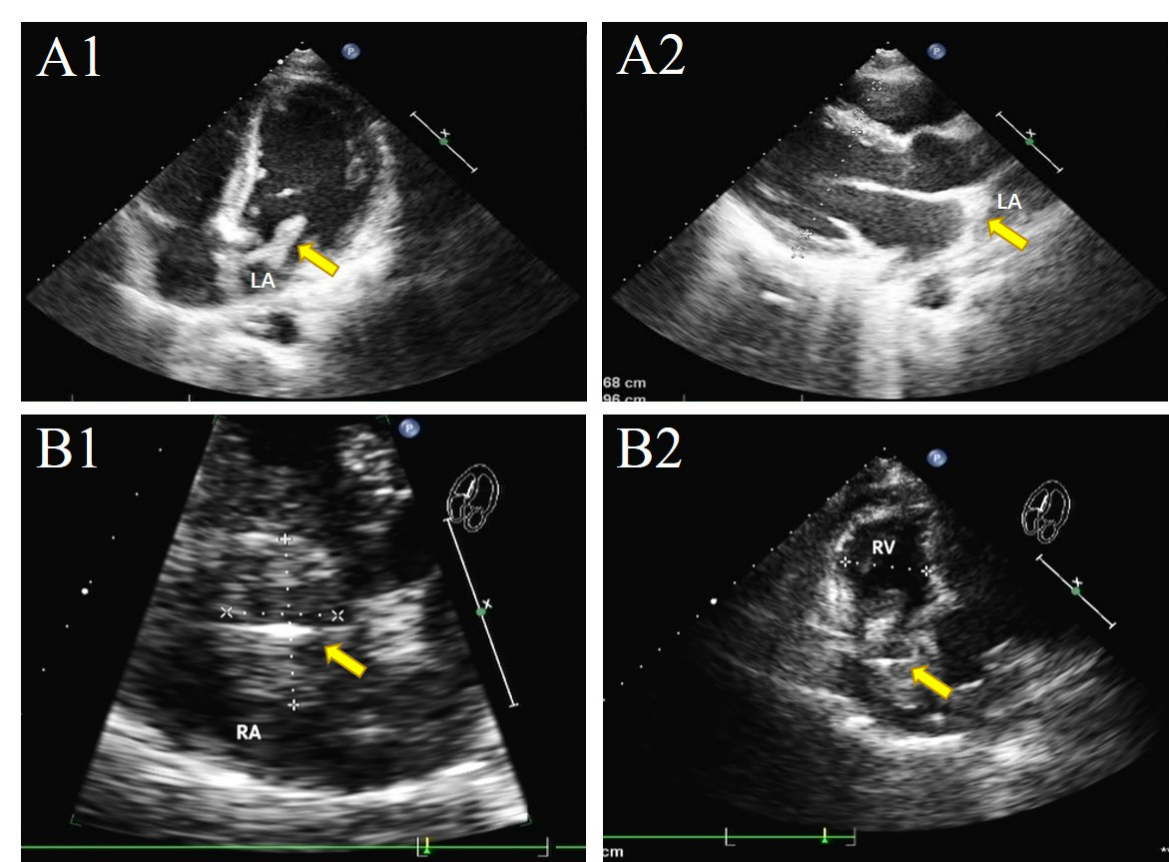
**TTE of IE with very large vegetations are susceptible to misdiagnosis**. Arrow: vegetations. (A1) The pedunculated vegetation in Case 1 protruded into the mitral valve orifice during diastole, (A2) and returns to the left atrium during systole. (B1) In Case 15, TTE showed fixed fuzzy echoes of the right atrium near the tricuspid valve orifice, surrounded by electrodes, (B2) and incarcerated in the tricuspid valve orifice.

**Fig. 5.**
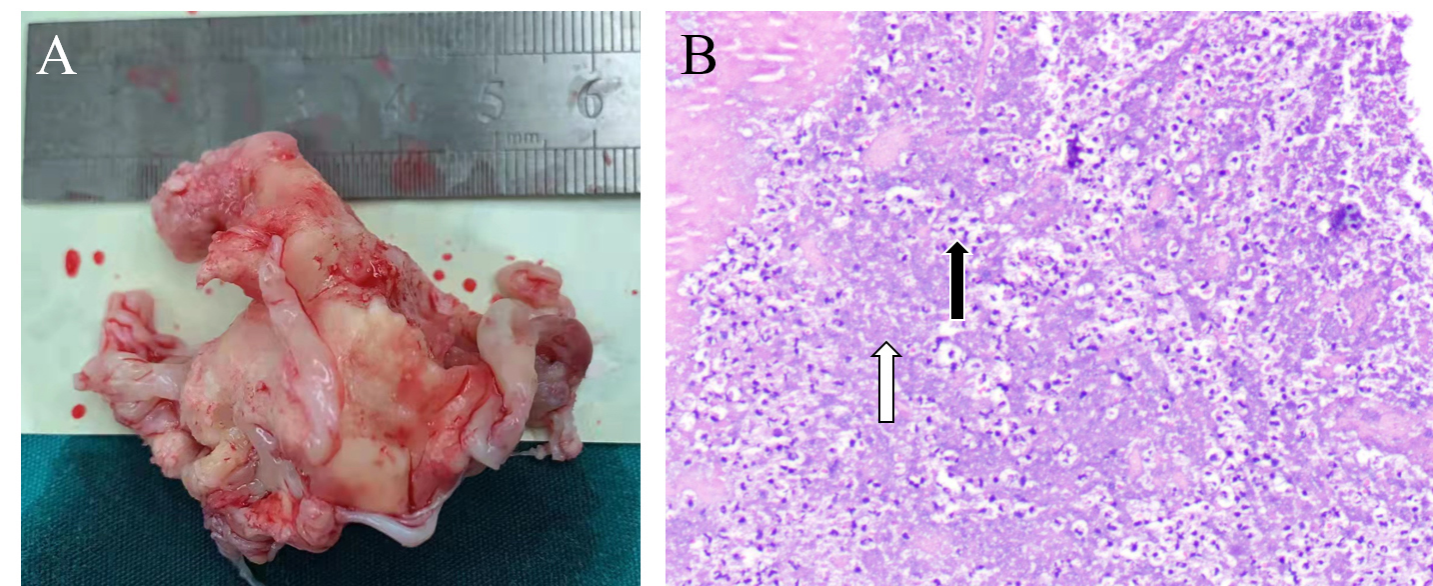
**Tricuspid valve vegetations in IE**. (A) The gross appearance of a large vegetation on the tricuspid valve in Case 16, as measured in centimeters. (B) Hematoxylin and eosin staining of a microscopic cross section of a vegetation. White arrow: bacteria embedded within the vegetation. Black arrow: inflammatory cells.

Diagnosis and management of IE remains challenging because of its clinical diversity and changing epidemiology [11, 12]. The disease pattern and prognosis of IE vary greatly due to pathogenic microorganisms, basic heart disease, implantation of prosthetic valves and cardiac devices, etc. Therefore, even risk factor classification in international guidelines is inconsistent with clinical practice [13, 14, 15]. In addition, there are only infrequent occurrence and sparse reports of IE with very large vegetations, so the original clinical evaluation can confound its diagnosis. Patients with atypical symptoms visit multiple departments. Less-experienced physicians may consider their diagnosis as chronic infection, rheumatic disease, neurological disease, autoimmune disease, malignant tumor, etc. [16, 17, 18]. Although TTE is a mainstay in the diagnostic toolkit for IE [19], sometimes it is difficult to differentiate one mass from another. Our findings suggested that, in IE patients with atypical clinical presentation, the mass in the left atrium is readily misdiagnosed as myxoma. In this study, Case 1 showed that the pedunculated vegetation seemed to be attached to the mitral valve annulus (Fig. 4). A similarly confusable state also existed in Case 2 with the vegetation located in the posterior mitral valve and in Case 3 with the pedicle of vegetation attached to the junction of the anterior and posterior mitral valves. These were originally considered as left atrial myxoma and were planned to undergo tumor enucleation. During the operation, the surgical plan was changed to vegetectomy and mitral valve replacement. Finally, the postoperative biopsy confirmed the diagnosis of IE. A mass in the right atrium might be wrongly diagnosed as thrombosis. The TTE of Case 16 showed tricuspid septal fixed irregular hypoechoic mass, and the TTE of Case 15 showed fixed fuzzy echoes of the right atrium, surrounded by electrodes incarcerated in the tricuspid valve orifice (Fig. 4). The TTE diagnosis of Case 15 and Case 16 were high possibility of thrombosis, but postoperative pathology confirmed IE (Fig. 5). The blood test results of 22 patients in this study are consistent with the authoritative review [20], which points out that an IE diagnosis may be suspected due to elevated CRP, ESR, anemia, and microscopic hematuria, etc. However, the above indicators lack specificity and have not been included in modern clinical diagnostic criteria. The negative blood culture rate of 31.8% (7/22) in our IE cases with >30 mm vegetation seems to be higher than previous reports of those <30 mm [2, 21, 22, 23]. This may be due to previous antibiotic treatment, microbes embedded deep within the giant vegetation and not released into the blood, and the relatively small study sample size. We prefer initial treatment with AMP and CRO [24], and CRO combined with TZP, and VAN combined with CRO are reasonable empirical treatments [25, 26, 27].

In conclusion, in terms of improving early diagnosis, our study suggests that IE accompanied by giant vegetation has a wide variety of first appearances, diverse imaging features, dangerous clinical condition, and a high negative blood culture rate. Clinicians should be more vigilant about IE when a large space-occupying mass is found by TTE. High suspicion of IE in the early stage of clinical admission is appropriate for patients with the following manifestations. (a) With underlying structural heart disease or with intracardiac implants, (b) with vascular manifestations such as arterial embolism, pulmonary embolism, and intracranial hemorrhage, (c) with consumptive disease manifestations such as anemia and hypoalbuminemia, (d) with unexplained manifestations of acute heart failure, (e) with fever and arterial embolism. Patients with unexplained spleen, brain, or kidney infarction as the first symptom should also be alerted to the possibility of IE.

**Fig. 6.**
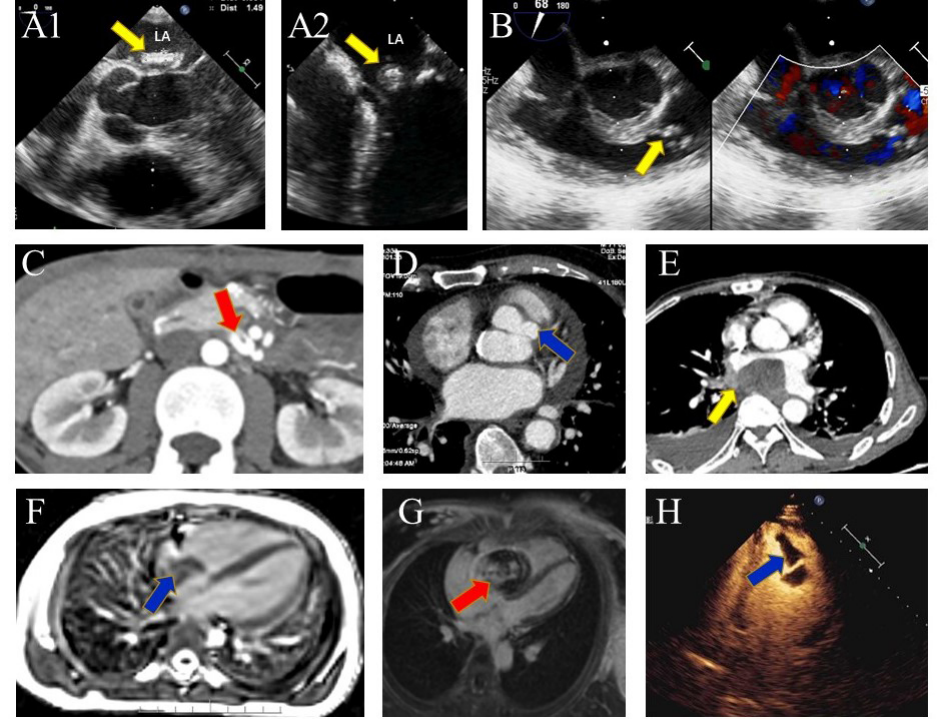
**Multimodality imaging in IE**. (A) TEE showed a very large mitral valve vegetation in the left atrium. Yellow arrows: vegetations. (A1) TEE of Case 2 showed a pedicle connection to the mitral valve rather than the atria, which was confirmed to be IE by biopsy. (A2) Left atrial vegetation of Case 6. (B) TEE demonstrates pulmonary valve vegetations in a Case of patent ductus arteriosus. (C) Aortic CTA shows superior mesenteric artery aneurysm. Red arrow: rupture of pseudoaneurysm in Case 6. (D) Cardiac CT revealed a perivalvular aortic abscess (blue arrow) of Case 16. (E) CT showing cardiac metastases (primary gastric inflammatory myofibroblastic tumor). Yellow arrow: uneven enhancement. (F) MRI shows abnormal signal foci in the right atrium, with no obvious enhancement after enhancement. Blue arrow: thrombus in the right atrium. (G) Most of the lesions did not enhance during delayed MRI enhancement. Red arrow: Myxoma. (H) Myocardial perfusion ultrasound showed two moderate echoic masses in left ventricle, and there was no contrast agent filling in the irregular masses. Blue arrow: thrombus.

Compared with the 10–20 mm vegetation group, the diagnosis of IE with very large vegetation is more difficult as it is often mixed with basic heart diseases or implantations. Compared with TTE, transesophageal echocardiography (TEE) can more accurately assess the hemodynamic effects of location, attachment site, size and shape of vegetation and valve damage (Fig. 6A1,A2,B), especially in the case of prosthetic valve endocarditis (PVE) and perivalvular abscess. However, cardiac computed tomography/computed tomography angiography (CT/CTA) is more suggestive in detecting perivalvular complications (abscess/pseudoaneurysm) in native valve endocarditis (NVE) and PVE, and non-cardiac abnormalities can be found in a single examination [28]. Case 6 was admitted to hospital due to abdominal pain for 9 days, with no fever and negative blood culture. TTE showed echo mass in the left atriun, and aortic CTA showed mesenteric artery pseudoaneurysm (Fig. 6D). Thrombosis and abscess were seen during aneurysmectomy, and MVR was performed after oral antibiotics for 2 weeks. Mitral valve biopsy was consistent with IE, so CTA was of great significance for prompting diagnosis of IE with atypical clinical features. Case 16 was admitted with negative blood cultures and a paravalvular aortic abscess detected by cardiac CT (Fig. 6D). CT can also be used to differentiate between benign and malignant tumors (Fig. 6E). Magnetic resonance imaging (MRI), on the other hand, can better differentiate tissue components (solid, liquid, hemorrhage, fat, and thrombus), and is especially useful in differentiating giant vegetations from thrombus (Fig. 6F) or myxomas (Fig. 6G). Myocardial perfusion ultrasonography is also useful in excluding thrombus (Fig. 6H). Because there are no lead artifacts in positron emission tomography (PET), its diagnostic ability for CDRIE is higher than CT. Silbiger and his coworkers [29] reported that PET reclassifies 90% of Duke-possible patients with suspected device infections, and PET was also superior in finding primary and extracardiac sepsis [30]. Doctors should be aware of the possibilities offered by the multimodal imaging approach when appropriate.

In addition to the emphasis on improving early diagnosis, our study also supports early surgical intervention in IE with >30 mm vegetations. Previous studies have shown that more than 80% of deaths from IE were associated with embolic events [31, 32]. In patients with giant vegetations included in the study, the proportions of previous and new embolism (within one week of antibiotic treatment) were significantly increased, which was consistent with the conclusion of many studies that the size of vegetations was related to the occurrence of embolism [33, 34]. The size of vegetation has been used to create an “embolism risk calculator” [35]. In a meta-analysis of 21 studies, patients with vegetation size greater than 10 mm had increased odds of embolic events (OR [Odds Ratio], 2.28), but odds were greater with a cutoff of 15 mm (OR, 4.25) [31, 36]. However, due to the rarity of giant vegetations, a 10 mm size of vegetations was considered to be the best threshold for estimating the risk of embolism in previous studies. There have been no reports on the relative risk of embolism for vegetations >30 mm or providing outcome data for further treatment until now.

There are few reported cases of giant IE vegetations worldwide. Radcliffe *et al*. [2] summarized all twenty-three IE cases with >40 mm vegetations reported before 2020, and we retrieved seven published IE cases with >30 mm vegetations published after 2020 (Table 6, Ref. [37, 38, 39, 40, 41, 42, 43]). In these 30 published cases, the vegetation was most commonly located in the tricuspid valve. By contrast, in the 22 patients we included, mitral valve vegetations were predominant, which may be attributable to publication bias and small sample size. Surgical intervention was performed in 69.9% (16/23) cases reported before 2020, with an overall mortality rate of 43%. Of the 22 cases we included and the seven cases of giant vegetations published after 2020 that we retrieved, 96.6% (28/29) underwent surgery, there were no cases of in-hospital mortality, and tricuspid regurgitation worsened postoperatively in only one case. These results seem to suggest the important role of early surgery for giant vegetations. Research has confirmed that antibiotic treatment alone was unsatisfactory for very large IE vegetations. IE vegetations >10 mm in TTE were associated with significantly lower response rates to specific medical therapy, and 65% of embolisms occurred within two weeks of antibiotic therapy [44, 45]. So despite antibiotic therapy, an increase in vegetation size was not unusual, predicting subsequent embolism [46]. However, the long-standing controversy over the surgical indications and timing in IE with >30 mm vegetations has not been resolved, which is reflected in the difference between the two authoritative guidelines [16]. ESC recommends urgent surgery for vegetations >30 mm to prevent embolism, but the timing is stipulated within a few days after diagnosis, and the evidence grade is IIa B. The ACC/AHA Guideline uses 10 mm as the dividing line, and does not specifically mention extremely large vegetation cases, with the suggested operation time prior to stopping antibiotics [47]. In fact, surgical indications are narrowed and surgical timing is delayed in clinical practice, as very large vegetations without comorbidities are rarely used as surgical indications [48]. Surgical treatment of IE is often performed after complications have occurred, rather than prophylactically to reduce the risk of future complications [16, 17, 49]. Meanwhile, serious complications are overanxiously considered as surgery contraindications, such that antibiotic therapy under close monitoring in the presence of cardiac function class III or IV is recommended. On the one hand, early surgery is crucial to reduce the risk of additional complications or clinical deterioration [50], and on the other hand, surgical urgency is strongly associated with higher in-hospital mortality [7, 51, 52]. However, more and more recent research supports early surgery (within one week after diagnosis) for IE patients with complex complications [53, 54, 55], and indicates aggressive surgical treatment is associated with better survival (HR = 0.58) and reduced 6-month mortality (11% for early surgery vs. 33% for delayed surgery, OR = 0.18) [56, 57]. A study assigned 76 IE patients with >10 mm vegetations and severe valve dysfunction to either early surgery within 48 hours or antibiotic therapy, concluding that early surgery prevented any additional embolic events and did not increase mortality [58].

**Table 6.** **Summary of IE Reports with >30 mm vegetations 2020 to 2022**.

Year/Location	Age/Sex	Size of Vegetation (mm)	Pathogen	Area of involvement	Antimicrobial Therapy	Length of antimicrobial Therapy	Surgery	Outcome
2020/USA [37]	27/F	34 × 20	MSSA, *S. marcescens*	TV	VAN/TZP	6 wk	Transcatheter aspiration	Success
2020/USA [38]	27/M	35 × 13	MRSA	TV	NR	8 d	Percutaneous Debulking of Vegetation	Worsening of TR
2020/USA [39]	55/M	50 × 20	MSSA	TV	NR	8 wk	CIED extraction + transcatheter aspiration + PE	Success
2020/USA [40]	54/M	39 × 9	No growth	TV	CRO	8 wk	AVR + TVR	Success
2021/Iran [41]	58/M	31 × 22	No growth	PV	CZO/TEC	7 wk	None	Success
2021/USA [42]	31/M	50 (length)	MRSA	TV	NAF	6 wk	TVP	Success
2022/Belgium [43]	61/M	45 (length)	MSSA	ATVL	CZO	6 wk	TVP	Success

Abbreviations: NR, not reported; MRSA, methicillin-resistant *staphylococcus aureus*; MSSA, methicillin-sensitive *staphylococcus aureus*; VAN, vancomycin; TZP, piperacillin-tazobactam; CRO, ceftriaxone; CZO, cefazolin; TEC, teicoplanin; NAF, nafcillin; CIED, cardiac implantable electronic device; PE, pulmonary embolism; AVR, aortic valve replacement; TVR, tricuspid valve replacement; TVP, tricuspid valve repair; TR, tricuspid regurgitation.

In our study, patients with >30 mm vegetation almost all underwent surgery before the end of a course of antibiotics. On the day of admission, we completed the medical history, physical examination, TTE, TEE and blood culture, evaluated and confirmed within 12 hours. Once we found large vegetations or suspected IE, we tried to improve cardiac and even whole-body imaging with CT/CTA, MRI, and so on. We discussed and addressed with a multidisciplinary team, and started anti-infective treatment promptly. Patients with >15 mm vegetations, heart failure, and paravalvular abscess need surgical intervention immediately or within 48 hours (Grade 1a, Grade B). Cardiac glycoside and diuretics were given before surgery to improve cardiac function. All patients underwent surgery on cardiopulmonary bypass. The surgeon completely removed vegetations and infected tissues, repaired or replaced damaged valves, and corrected intracardiac deformities. The standard operation technique was performed according to 2016 American Association for Thoracic Surgery (AATS) guidelines [59]. Surgical specimens were sent for pathogen culture and biopsy. Cardiac and renal function support and pharmacological anticoagulation were given after the operation. TTE was reviewed to evaluate valve function and complications. Antibiotics were administered for at least 4 weeks postoperatively. If the culture results of pathogens were negative, broad-spectrum antibiotics (VAN and aminoglycosides) were preferred.

In terms of the perioperative period, surgical timing, surgical method, details and prognosis, we summarized some experiences of IE with >30 mm vegetation that is different from normal-sized vegetations (Table 7).

**Table 7.** **Comparison of surgical treatment and prognosis between patients with vegetations >30 mm and 10–20 mm**.

	>30 mm	10–20 mm
Surgery within 1 week of admission (cases)	16	20
The number of valve replacements	24	23
The number of biological valves	9	4
Postoperative embolism/shock/ICH	10	2
Surgical reintervention	2	2
The number of patients with ICU-LOS ≥3 d	8	2
Death/recurrence	0	2

(1) In terms of preoperative examination, although routine brain imaging screening is reasonable in patients with left-sided IE (IIa B), the 2016 AATS consensus guidelines emphasizes the necessity of preoperative cranial examination in IE with very large vegetations. Because >30 mm vegetations often involve higher misdiagnosis rate and more serious infection, multimodality imaging is more emphasized to confirm the diagnosis and to find other infectious lesions or abscesses [60]. Coronary arteriography (CAG) in >30 mm aortic vegetations or aortic abscesses theoretically increases the risk of embolism, so CTA should be used instead of CAG (1 C).

(2) Because patients with >30 mm vegetations often have poor cardiac function, preoperative cardiotonic and diuretic treatments are more commonly used. However, caution should be taken with regard to the complex basic cardiac conditions in these patients, such as HCM and atrioventricular block.

(3) As for the timing of surgery, the decision of a multidisciplinary team is particularly significant in patients with >30 mm vegetations. A >30 mm vegetation with imminent embolism is an indication for surgery within 48 hours (IIa B), while normal-sized vegetations with new embolism despite reasonable antibiotic treatment or with severe valve disease should be performed with sub-emergency operation (IIa B). But in patients with >30 mm vegetations, if there is a strong indication for early surgery (hemodynamic instability or high risk of new embolic stroke), early surgery may be considered after careful evaluation of the size of the bleeding lesion by the multidisciplinary team [61, 62]. The risk of postoperative neurological deterioration due to ICH appears to be relatively low, even in IE patients who undergo valve surgery within 2 weeks after ICH [63], but very early surgery (within 7 days) should be avoided [64].

(4) The choice of surgical incision and the surgical difficulty are different between the 10–20 mm group and the >30 mm group. Compared with one case of minimally invasive MVR in the 10–20 mm group, median sternotomy was used in all patients with >30 mm vegetations. Our research suggests that aggressive S. aureus, CIED and PVE are more frequent in patients with >30 mm vegetations. Median sternotomy is beneficial for radical debridement and for more difficult operations, such as Bentall if the aortic root is badly damaged [65].

(5) According to 2016 AATS Guidline, when valvular stenosis and regurgitation occur, MVP is the optimal choice instead of MVR, while AVR is better than aortic valve plasty (AVP). Generally speaking, TVP is recommended. But in the patients with >30 mm vegetation, TVR in more common than in the 10–20 mm group (4 cases vs. 1 cases), and a biological valve is mostly used. This may be because the >30 mm vegetations group had more faulty TV, ICH, or severe stroke, so that it was reasonable to avoid mechanical prostheses, and the use of bioprosthetic valves could avoid postoperative anticoagulation required to reduce other hemorrhagic complications. The selection of valve type should consider the surgical difficulty that the biological valve may need to be completely replaced in case of IE relapse [66].

(6) According to the 2015 ESC IE Guidelines [67], as predictors of poor prognosis in admission assessment, compared patients with 10–20 mm vegetations, those with >30 mm vegetations had more complex clinical situations (such as pulmonary edema, pulmonary embolism, poor pulmonary function, cerebral infarction, acute heart failure, shock, and more diverse pathogens). There are many basic conditions in the heart in the >30 mm vegetations group (such as cardiomyopathy, pulmonary hypertension, history of heart surgery, prosthesis valves, intracardiac devices and low ejection fraction). The collaborative management of a multidisciplinary team and personalized treatment plan are critical to the outcome of IE patients with >30 mm vegetations. Our study supports that early surgery for IE patients with very large vegetations can reduce in-hospital and 6-month mortality, but we need better risk stratification in view of more postoperative cardiac tamponade, DIC, and ICH in patients than in the normal-sized vegetations group [58, 68, 69].

## 5. Conclusions

For IE complicated with >30 mm vegetations, clinical characteristics are diverse and vegetations on TTE are prone to misdiagnosis as thrombus or tumors. This article emphasizes the use of >30 mm IE vegetations as an independent indication for early surgery to improve prognosis.

## Abbreviations

AATS, American Association for Thoracic Surgery; APTT, activated partial thromboplastin time; AMP, ampicillin; AVP, aortic valve plasty; CAG, coronary arteriography; CRE, carbapenem-resistant* Enterobacter*; CDRIE, cardiac device-related infective endocarditis; CRTD, cardiac resynchronization-defibrillator device; cTn I, cardial troponin I; CKD5, chronic kidney disease stage 5; CHD, congenital heart disease; CRP, c-reactive protein; CT/CTA, computed tomography/computed tomography angiography; CK, creatine kinase; CK-MB, creatine kinase MB; DCM, dilated cardiomyopathy; DBIL, direct bilirubin; DIC, disseminated intravascular coagulation; DAA, double aortic arch; ESR, erythrocyte sedimentation rate; EuroSCORE II, european System for Cardiac Operative Risk Evaluation II; FDP, fibrinogen degradation product; GAS, group A streptococci; GBS, group B streptococci; HCM, hypertrophic cardiomyopathy; IE, infective endocarditis; ICU-LOS, intensive care unit length of stay; ICH, intracerebral hemorrhage; LDH, lactate dehydrogenase; LOS, length of stay; LY%, lymphocytes percentage; MRI, magnetic resonance imaging; MRSA, methicillin-resistant *staphylococcus aureus*; MSSA, methicillin-sensitive *staphylococcus aureus*; NE%, neutrophils percentage; NT-proBNP, N-terminal pro-brain natriuretic peptide; NVE, native valve endocarditis; TEE, transesophageal echocardiography; TZP, piperacillin-Tazobactam; PCT, procalcitonin; PET, positron emission tomography; PT, prothrombin time; PVE, prosthetic valve endocarditis; PE, pulmonary embolism; SP, severe pneumonia; Q1–Q3, the 25th–75th percentiles; ACC, the American College of Cardiology; ESC, the European Society of Cardiology; TBIL, total bilirubin; TTE, transthoracic echocardiography; VAN, vancomycin; VVI, ventricular demand pacemaker; WBC, white blood cells.

## Supplementary Material

Supplementary material associated with this article can be found, in the online version, at https://doi.org/10.31083/j.rcm2308264.
