# Supplementary material for: Analysis of Clinical Features and Outcomes of Infective Endocarditis with Very Large Vegetations: A Retrospective Observational Study from 2016 to 2022
Source: Rev Cardiovasc Med. 2022 Jul 21;23(8):264. doi: 10.31083/j.rcm2308264 (PMC11266946; doi:10.31083/j.rcm2308264)
Supplement: Supplementary file 1 [file 2153-8174-23-8-264-s1.doc]

**Supplementary Table 1. Comparison of laboratory variables in blood tests between patients with >30 mm vegetations and those with 10–**20 mm vegetations.

| Laboratory variables | Preoperative value in >30 mm group | 10–20 mm group | *p* value (vs. 10–20) |
| --- | --- | --- | --- |
| WBC count (× 10^9^/L) | 9.4 (7.4–13.4) | 7.9 (6.7–10.0) | 0.116 |
| Hemoglobin (g/L) | 89.5 (72.0–98.5) | 102.0 (78.8–119.3) | 0.108 |
| Platelets (× 10^9^/L) | 191.0 (97.5–276.3) | 208.0 (165.3–230.3) | 0.907 |
| NE (%) | 80.2 (69.6–87.5) | 76.3 (61.8–82.9) | 0.231 |
| LY (%) | 15.5 (8.9–18.5) | 14.8 (8.8–26.0) | 0.453 |
| CRP (mg/L) | 61.9 (32.3–83.4) | 47.5 (12.2–74.1) | 0.118 |
| ESR (mm/h) | 67.0 (34.8–82.5) | 64.0 (51.0–105.0) | 0.885 |
| APTT (s) | 31.8 (22.6–29.5) | 33.8 (31.6–36.9) | 0.218 |
| Albumin (g/L) | 31.2 (27.9–35.1) | 33.8 (27.2–37.0) | 0.467 |
| TBIL (μmol/L) | 12.8 (9.5–17.9) | 10.1 (7.5–13.1) | 0.136 |
| DBIL (μmol/L) | 6.1 (4.2–10.7) | 5.3 (3.9–6.9) | 0.113 |
| Creatinine (μmol/L) | 111.5 (78.5–128.0) | 75.5 (64.2–92.3) | 0.565 |
| LDH (U/L) | 336.0 (217.7–403.5) | 255.0 (226.0–289.0) | 0.176 |
| Total protein (g/L) | 74.7 (62.4–74.7) | 66.8 (61.1–74.2) | 0.231 |
| PT (s) | 13.8 (12.9–14.9) | 13.9 (12.9–15.6) | 0.991 |
| Urea (mmol/L) | 4.4 (2.8–9.1) | 4.8 (3.4–7.5) | 0.963 |
| NT-proBNP (pg/ml) | 1686 (966.1–3535.0) | 767.0 (203.0–7640.0) | 0.297 |
| ALT (U/L) | 23.6 (15.3–36.5) | 33.1 (10.6–52.0) | 0.581 |
| AST (U/L) | 24.6 (18.0–39.6) | 28.2 (16.3–41.0) | 0.742 |
| CK (U/L) | 28.9(26.7–59.0) | 29.8 (19.2–57.0) | 0.509 |
| CK-MB (U/L) | 11.4 (7.2–16.9) | 8.1 (6.0–12.4) | 0.468 |
| PCT (ng/ml) | 1.5 (0.2–2.4) | 0.1 (0.0–0.3) | 0.002* |
| D dimer (mg/L) | 0.7 (0.4–1.4) | 0.4 (0.2–0.6) | 0.040* |
| cTn I (ng/mL) | 0.1 (0.0–0.2) | 0.0 (0.0–0.0) | 0.059 |

**p* < 0.05.
